# Environmental adaptation and vertical dissemination of ESBL‐/pAmpC‐producing *Escherichia coli* in an integrated broiler production chain in the absence of an antibiotic treatment

**DOI:** 10.1111/1751-7915.13040

**Published:** 2018-01-17

**Authors:** Michaela Projahn, Katrin Daehre, Torsten Semmler, Sebastian Guenther, Uwe Roesler, Anika Friese

**Affiliations:** ^1^ Institute for Animal Hygiene and Environmental Health Freie Universität Berlin Berlin Germany; ^2^ Robert Koch‐Institute Berlin Germany; ^3^ Present address: German Federal Institute for Risk Assessment, Diedersdorfer Weg 1, D‐12277 Berlin Germany

## Abstract

High prevalence numbers of extended‐spectrum beta‐lactamase‐ (ESBL‐)/plasmid‐mediated AmpC beta‐lactamase‐ (pAmpC‐) producing *Escherichia coli* in broiler chicken and their distribution along the broiler production chain is an ongoing problem in food production. We, therefore, investigated resistant isolates along the broiler production chain to determine whether there is a constantly occurring direct vertical transmission of the ESBL‐/pAmpC‐producing *E. coli* from the parent flocks to their offspring or not. We, furthermore, analysed the isolates concerning the occurrence of virulence factors and their ability to form biofilms to estimate their potential to effectively colonize broiler chickens and/or persist and survive in the environment of the broiler production facilities. Using whole genome sequencing, we could show that ESBL‐/pAmpC‐producing *E. coli* were likely transferred in a step‐wise process along the broiler production chain but not directly from the parent flock to the fattening flock with every single batch of offspring chickens. Additionally, resistant *E. coli* strains showing an extraintestinal pathogenic genotype as well as high numbers of virulence‐associated genes including the production of curli fibres and cellulose have high capabilities to persist and spread in the broiler production chain.

## Introduction

Extended‐spectrum beta‐lactamase‐ (ESBL‐) and plasmid‐mediated AmpC beta‐lactamase‐ (pAmpC‐) producing Enterobacteriaceae are an increasing problem in public health and veterinary medicine (Pitout and Laupland, 2008; Ewers *et al*., 2012; Kaesbohrer *et al*., 2012). A major concern is the high prevalence of these resistant bacteria in the broiler production chain. ESBL‐/pAmpC‐producing Enterobacteriaceae were frequently isolated from broiler fattening farms worldwide (Bortolaia *et al*., 2010; Randall *et al*., 2011; Kameyama *et al*., 2013; Maciuca *et al*., 2015; Trongjit *et al*., 2016) but were also found in broiler (grand) parent flocks (Dierikx *et al*., 2013; Agersø *et al*., 2014; Mo *et al*., 2014; Zurfluh *et al*., 2014). Therefore, different transmission scenarios of the resistant bacteria in the broiler production pyramid are discussed: A transmission from prior stages into the fattening farms (Giovanardi *et al*., 2005; Dierikx *et al*., 2013; Nilsson *et al*., 2014; Huijbers *et al*., 2016) as well as an insufficient cleaning and disinfection procedure in the chicken barns (Hiroi *et al*., 2012; Luyckx *et al*., 2015b). We recently showed a pseudo‐vertical transfer of resistant bacteria from the parent flocks into the hatchery via contaminated eggshells (Projahn *et al*., 2017). In addition, it was proved by whole genome analyses that there is in fact a horizontal transfer of ESBL producers between consecutively fattened flocks regardless of cleaning and disinfection procedures (Daehre *et al*., 2017). However, it still remained unclear whether the resistant bacteria are permanently and repeatedly transferred along the whole broiler production chain with each production cycle, or if this transfer only occurs occasionally and certain strains, once introduced on the farm, are circulating constantly.

*Escherichia coli* naturally inhabits the gastrointestinal tract of mammals and birds and can be classified into commensal and pathogenic strains. Intestinal pathogenic *E. coli* (IPEC) are causing diarrhoea syndromes and seldom colonize healthy humans whereas extraintestinal pathogenic *E. coli* (ExPEC) often innocuously colonize the gut but have the ability to cause severe infections such as meningitis or blood stream infections (Vila *et al*., 2016). Recent studies compared the occurrence of certain virulence‐associated genes (VAGs) in avian pathogenic *E. coli* (APEC), uropathogenic *E. coli* (UPEC) and commensal *E. coli* from humans and broiler chickens (Kemmett *et al*., 2013; de Carli *et al*., 2015; van Hoek *et al*., 2016; Paixao *et al*., 2016; Silveira *et al*., 2016). It turned out that certain VAGs (e.g. *fimC, iha, tsh, ireA, neuC, astA, irp2, vat, iucD, chuA, iss)* could not be statistically significant linked to a particular type of pathogenicity, but were also detected in strains from healthy hosts. Therefore, some of these factors are increasingly termed as ‘fitness factors’ because they contribute to a successful colonization and enhanced survival in the gut and the environment but not necessarily cause diseases (Smith *et al*., 2007; Frommel *et al*., 2013; Wigley, 2015; Vila *et al*., 2016). ESBL‐/pAmpC‐producing *E. coli* from healthy hosts were usually classified as commensal strains but recent investigations found out that these resistant strains also show characteristics of ExPEC or ExPEC‐like strains and, therefore, have the ability for an enhanced colonization of the gut.

The presented study aimed three questions. First, is there a direct top‐down transmission of ESBL‐/pAmpC‐producing *E. coli* from the parent flock via the hatchery to the respective fattening flock in the absence of an antibiotic treatment? Second, is the prevalence of ESBL‐/pAmpC‐producing *E. coli* in healthy broiler flocks influenced by their status as an ExPEC? Third, have the isolates found in the broiler production chain a higher ability to persist and survive in the environment?

To investigate the top‐down transmissions across all stages of the broiler production chain (parent flock, hatchery, fattening flock/barn), we analysed 44 ESBL‐/pAmpC‐producing *E. coli* from two different broiler production chains (chain C and F) which were not treated with antibiotics by whole genome analyses (Daehre *et al*., 2017; Projahn *et al*., 2017). Ten ESBL‐/pAmpC‐positive strains from parent screening samples (S) and other parent or fattening flocks (A/11, B/41, D, E/73) showing the same phylogroup and resistance genes that were found in chain C or F, respectively, were also included to show a potential circulation of certain strains within the broiler production chain (please see Fig. 4 and Table S1 for assignment/glossary of samples and strains). Secondly, to address the question whether there is a difference in the VAGs related to ExPEC between high and low prevalent strains, 89 selected ESBL‐/pAmpC‐positive strains from three healthy broiler flocks (C/41, E/74, F/74) with different prevalence were analysed for their VAGs. In addition, to determine the potential to persist and survive in the environment, the expression of two major biofilm‐associated extracellular matrix components (cellulose, curli fimbriae) was investigated for 13 strains from these three flocks.

## Results

### Phylogenetic analyses

*In silico* multilocus sequence typing (MLST) using whole genome sequences revealed three different sequence types (STs). Isolates, previously determined as phylogroup F harbouring a *bla*_CMY‐2_ gene and sampled from the broiler chain C were assigned as ST‐354. Samples from parent flocks D and screening parent flock S were also of ST‐354 whereas isolates of the fattening flocks A/11 and B/41 turned out to be of ST‐38. All isolates of phylogroup F harbouring a *bla*_SHV‐12_ gene of broiler chain F as well as from the additional samples belonged to the ST‐117.

**Figure 1:**
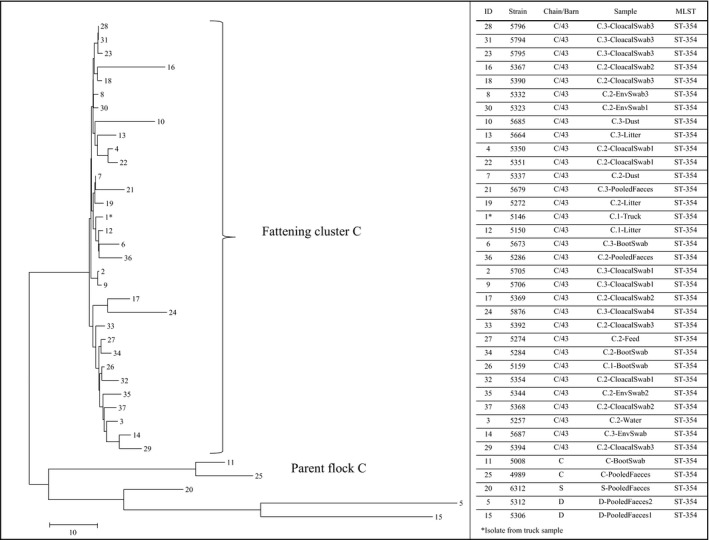
Phylogenetic tree of ST‐354 strains. A neighbour‐joining tree was calculated (with 1000 bootstraps) using a SNP distance matrix calculated by MEGA7 and the Harvest suite. Isolates are indicated by the respective strain number and the sample from which they originate as well as the chain and barn number of the respective sampling. The sample name presents further information like the sampling time points (C.1 – arrival of the chicks at the farm, C.2 – middle of the fattening period, C.3 – end of the fattening period) and the sample matrix.

**Figure 2:**
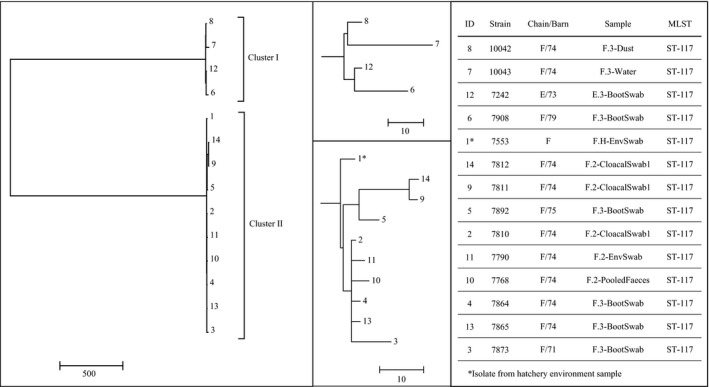
Phylogenetic tree of ST‐117 strains. A neighbour‐joining tree was calculated (with 1000 bootstraps) using a SNP distance matrix calculated by MEGA7 and the Harvest suite. Isolates are indicated by the respective strain number and the sample from which they originate as well as the chain and barn number of the respective sampling (E/73, F/71, F/74, F/75, F/79). The sample name presents further information like the sampling time points (F.H – Hatchery, F.2 – middle of the fattening period of chain F, F.3 – end of the fattening period of chain F, E.3 – end of the fattening period of chain E which was fattened at the same farm previously to the chickens of chain F) and the sample matrix.

Comparative analyses of single nucleotide polymorphisms (SNPs) were carried out for isolates of the MLST types ST‐354 (*n* = 37) and ST‐117 (*n* = 14) respectively (Figs 1 and 2). The calculation of the number of SNP differences between the ST‐354 strains (*bla*_CMY‐2_) resulted in one large cluster (fattening cluster: 0‐32 SNPs difference, max. 6.4 SNPs Mbp^−1^), which included all the isolates from the fattening flock C/41 (*n* = 31) as well as the isolate 5146 from the transportation truck of the same flock (Fig. 1). The SNP differences between this fattening cluster and the two strains (5008, 4989) from the respective parent flock C were calculated as 53 and 78 SNPs (10.5 and 15.6 SNPs Mbp^−1^) respectively. The SNP differences between the fattening cluster and the samples from parent flock D and screening parent flock S were calculated as up to 118 SNPs (23.6 SNPs Mbp^−1^).

The SNP difference calculation of the ST‐117 strains revealed two clusters (Fig. 2). The first one includes only four isolates of which two were collected from samples of fattening flock F/74 (chain F) as well as two screening isolates of fattening flocks E/73 and F/79. The second cluster comprises the isolate 7553 from the hatchery environment (chain F), seven isolates of the fattening period of flock F/74 (chain F) as well as two isolates from other flocks fattened at the same farm at the same time as fattening flock F (barn 74) but in different barns (F/71, F/75). SNP differences among the strains of the clusters varied between 23 and 68 SNPs (max. 13.6 SNPs Mbp^−1^) and 4 to 51 (max. 10.2 SNPs Mbp^−1^) SNPs, respectively, whereas the differences between both clusters were determined as 3114 to 3147 SNPs (max. 629.4 SNPs Mbp^−1^).

### Virulence‐associated genes

Whole genome contigs of the 89 strains were also investigated for their virulence profiles analysing 89 VAGs (Table S3) of which 19 genes were typically associated with IPEC strains (InVAGs) and 70 with ExPEC strains (ExVAGs). All strains were negative concerning the occurrence of InVAGs. In contrast, of the 70 ExVAGs, 37 genes were detected in ST‐354 strains (53%; chain C, screening samples D+S), up to 25 genes in ST‐117 strains (36%; chain F, screening samples E/73, F/71, F/75, F/79) and in the isolates of ST‐38 (36%; samples A/11, B/41), respectively, and 19 ExPEC‐related VAGs in the ST‐2307 isolates (27%). All isolates of the different MLST types share 14 ExVAGs which were as follows: *crl* (curli fibre gene), *csgA* (curli fibre‐encoding gene), *feoA/B* (major bacterial ferrous iron transporter, iron (II) transport system), *fimC* and *fimH* (type 1 fimbriae), *matA* (meningitis‐associated and temperature‐regulated fimbriae), *iroN* (catecholate salmochelin receptor), *iss* (increased serum survival), *sitA* and *sitC* (*Salmonella* iron transport system gene), *cvi* (structural genes of colicin V operon), *ompA* (outer membrane protein), *traT* (transfer protein), *astA* (heat stable cytotoxin associated with enteroaggregative *E. coli*) and *malX* (pathogenicity‐associated island marker CFT073). Most variations between the isolates of the different MLST types were observed in the numbers of genes which contribute to adhesion and iron uptake (Table 1). Moreover, certain traits were exclusively found in isolates of a respective ST. *bfpM* (bundle‐forming pilus), *ibeA* (invasion of brain endothelium), the *pap* operon (Pap pili adhesin), *tia* (toxigenic invasion locus in ETEC isolates) and *neuC* (K1 capsular polysaccharide) were detected in isolates of the ST‐354 whereas *fyuA* (yersiniabactin receptor), *ireA* (iron‐responsive element), *irp2* (yersiniabactin synthesis) and *pic* (serine protease autotransporter) occurred only in ST‐117 strains. *hek/hra* (heat‐resistant agglutinin) could be detected in the three ST‐38 isolates from the parent flocks A and B, only. Even though all analysed isolates showed very high numbers of detectable VAGs, according to the definition of ExPEC strains by Johnson *et al*. (2003)(≥2 of *papA/C*,* afa/dra*,* sfa/foc*,* iutA* and *kpsMTII*), only the ST‐354 strains and two of three ST‐38 strains (3565, 10026) could be assigned as an ExPEC. Interestingly, ExPEC‐related invasion genes also occurred in ST‐354 and ST‐38 strains, only.

**Table 1:** Mean values of detected virulence‐associated genes (VAGs). Highly variable categories are highlighted in bold

MLST	ST‐354	ST‐117 (cluster 1)	ST‐117 (cluster 2)	ST‐2307	ST‐38
Total no. of ExVAGs (70;14^a^)	36.7	21	24.3	19	23.7
Adhesion (34;5^a^)	**15.9**	**4**	**5**	**5**	**7**
Invasion (4;0^a^)	2	0	0	0	1
Iron uptake (12;4^a^)	**8.8**	**8**	**11**	**6**	**8**
Protection (6;4^a^)	6	3	4	4	4.7
Toxins (12;1^a^)	3	3	3.3	3	2
Miscellaneous (2;1^a^)	1	2	2	1	1

a Number of genes which were detected in all isolates.

### Macrocolony assay

**Figure 3:**
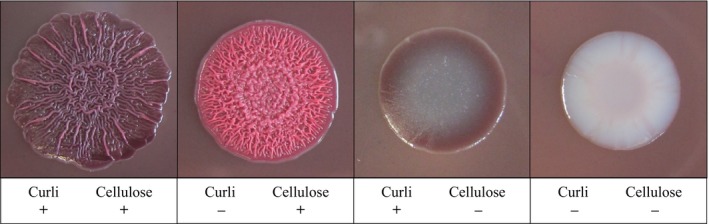
Macrocolony assay. Categories of detected phenotypes in the investigated isolates. The figure shows exemplary macrocolonies of isolates 6818 (+/+), 6922 (−/+), 3233 (+/−), 6985 (−/−).

Two sets of isolates were analysed by the macrocolony assay. The first one comprised thirteen isolates from the whole genome approach, at least three isolates of each MLST type (chain C: ST‐354, chain F: ST‐117, screening samples from A/11+B/41: ST‐38, Daehre *et al*. chain E: ST‐2307). Isolates of fattening flocks C/41, F/74 and E/74 occurred with different prevalence in the respective fattening flocks (flock C/41: 73.1%, flock E/74: 26.4%, flock F/74: 5.7%). The second strain set included 42 isolates from investigations of seven different broiler production chains covering all detected genetic profiles (phylogroup + ESBL‐/pAmpC gene) to get an overall impression on cellulose and curli production in different resistant isolates from the whole broiler production chain (Daehre *et al*., 2017; Projahn *et al*., 2017) (Tables S1 and S2). Isolates of the first set showed both, production of cellulose and curli, regardless of the flock prevalence, whereas in the second one, various phenotypes were determined (Fig. 3). In total, 28.6% (12/42) of the second strain set showed neither a production of curli nor cellulose, 7.1% (3/42) showed only a cellulose‐positive phenotype and 9.5% (4/42) of the isolates were positive for a curli production, only. Most of the strains (52.4%, 22/42) were phenotypically positive for both, curli and cellulose production. Only 16.7% (7/42) could not be assigned to a distinct phenotype in the macrocolony assay.

## Discussion

We performed our study addressing the questions of a direct top‐down transmission of ESBL‐/pAmpC‐producing *E. coli* from the parent flock via the hatchery to the respective fattening flock in the absence of an antibiotic treatment as well as the role of the ExPEC status and the ability to form biofilms on the prevalence of ESBL‐/pAmpC‐producing *E. coli* in healthy broiler flocks. Therefore, we investigated isolates (ST‐354) from a parent flock and the corresponding fattening flock (chain C) using whole genome analyses. It turned out that the isolates of the parent flock and the fattening flock are closely related but the grouping into different clusters and the analyses of the SNP differences showed that there was no direct vertical spread of these resistant bacteria. ESBL strains from the fattening period, collected during a time frame of five weeks, differed in not more than 6.4 SNPs Mbp^−1^. In contrast, isolates of the parent flock were collected only 17 days earlier but the SNP differences between isolates of the parent flock and the fattening flock were about 10.5 SNPs Mbp^−1^. The number of SNP differences between the strains of the fattening period is higher than the numbers published for clonal outbreaks of enterohemorrhagic *E. coli* (EHEC) (de Been *et al*., 2014; Berenger *et al*., 2015; Rusconi *et al*., 2016) but overall lower than those described for interspecies transmissions of clonal ST‐410 *E. coli* strains (Schaufler *et al*., 2016b). These findings suggest that ESBL‐/pAmpC‐resistant *E. coli* were more likely transferred from the parent flock into the production chain via an earlier event but not directly along the investigated batch of broiler chicken.

In addition, we isolated an ESBL‐*E. coli* strain from the transportation truck and from the litter inside of the barn from the first day of the fattening period which clustered together with all the other strains from the whole fattening period of chain C. This indicates that a transmission of the resistant bacteria can also occur via the transportation process and that the resistant bacteria must have been already present in the hatchery than directly transmitted from the parent flock. This is in concordance with our findings that the resistant bacteria were introduced into the hatchery via contaminated eggshells and the hatchery acts as a bottleneck for the spread of these strains (Projahn *et al*., 2017).

Investigation of the ST‐117 strains of chain F underlines the fact that there were ESBL *E. coli* already present in the hatchery which lead to the colonization of the recently hatched chicks. Whole genome analyses revealed two clusters. The first one comprised strains from the environment of the hatchery and the fattening period confirming the hatchery as a contamination source. The second ST‐117 cluster comprised strains of the fattening flock F as well as strains of two other barns from the same farm. Due to the small number of SNP differences between the isolates, a transmission or exchange of these ESBL *E. coli* between the barns of the same farm is very likely. It was previously shown that (ESBL‐) *E. coli* can survive the cleaning and disinfection procedures in the chicken barns which finally leads to a spread and the colonization of broiler chickens on the same farms (Luyckx *et al*., 2015a,b; Daehre *et al*., 2017). Interestingly, the two clusters differ in up to 629.4 SNPs Mbp^−1^ indicating that two different clonal lines of ST‐117 strains were introduced into the same flock.

We previously investigated up to 150 samples from the fattening period of seven flocks each showing that there is an overall highly diverse *E. coli* population on these fattening farms (Daehre *et al*., 2017). Together with the results from this study, we could show that not only one prominent vertical or horizontal transmission route of ESBL producers exists. More precisely, certain ESBL‐/pAmpC‐producing *E. coli* seemed to be more likely introduced into the broiler production chain decades ago and now circulate constantly in different broiler production‐related environments and evolve even without antibiotic pressure (Nilsson *et al*., 2014; Huijbers *et al*., 2016; Mo *et al*., 2016).

Investigations on the phenotypic characteristics and virulence patterns of ESBL‐/pAmpC‐producing *E. coli* indicate that certain traits usually associated with virulence factors correlate with the ability to adhere, persist and adapt to certain environmental circumstances (Guenther *et al*., 2012; Rodiger *et al*., 2015; Mo *et al*., 2016; Paixao *et al*., 2016; Schaufler *et al*., 2016a). In our study, high prevalence strains of ST‐354 could be assigned as ExPEC and overall showed the highest number of ExVAGs, especially genes referring to adhesion factors, compared to low prevalent strains of ST‐117 and ST‐2307. ST‐354 isolates harboured the *pap* operon which was shown to be responsible for the *in vitro* adhesion to not only human but also animal cell lines (Stromberg *et al*., 1990; Frommel *et al*., 2013). But intestinal colonization is also depending on the iron uptake ability of *E. coli* (Nowrouzian *et al*., 2006; Tenaillon *et al*., 2010). On the one hand, ST‐117 strains of our study had the highest number of genes referring to iron uptake, on the other hand, these isolates had low numbers of genes which contribute to adherence factors. Furthermore, these strains were found with low prevalence in the investigated fattening flock F (5.7%). Interestingly, ST‐2307 isolates had overall the lowest numbers of ExVAGs including adherence traits but occurred with higher prevalence in the fattening flock (23.5%) than ST‐117 strains assuming that not only high numbers of VAGs but a certain combination or set of virulence traits can promote the colonization of the avian gut. However, specific factors for the binding to avian host cells are poorly understood and should be therefore further investigated.

The ability to form bacterial biofilms is a benefit in the survival against host defence factors, antibiotics, physical and chemical stress as well as disinfectants (Hall‐Stoodley *et al*., 2004; Flemming and Wingender, 2010). Using a macrocolony assay, we could show that more than half of our tested strains from both investigated sets were able to produce curli fibres and the exopolysaccharide cellulose which are necessary to form stable and strong biofilm matrices (Olsen *et al*., 1989; Barnhart and Chapman, 2006; Uhlich *et al*., 2006; Serra *et al*., 2013, 2015). These positive strains were isolated from all the seven investigated flocks by Daehre *et al*. and included also the 14 selected isolates from the whole genome analyses of ST‐354, ST‐117, ST‐38 and ST‐2307 from our study. Here, the production of curli fibres and cellulose was not linked to a certain phylogroup or ESBL/pAmpC resistance gene and was also independent from the prevalence of these strains in the respective flocks (flock C: 73.1%, flock E: 26.4%, flock F: 5.7%) indicating that resistant *E. coli* occurring on broiler farms in general show good abilities to survive on the farms and in the environment. Cellulose also has high water holding capacities which protects the bacteria against dehydration (O'Sullivan, 1997). This could be a big advantage in surviving, for example, the cleaning and disinfection procedure in the hatchery and the broiler fattening farms. Daehre *et al*. already showed that the ST‐2307 strains were transmitted from one broiler flock to the consecutively fattened flock in the same barn. The results of the present study suggest that biofilm production as well as high numbers of gene conferring to adherence might support the persistence of resistant bacteria and the stable colonization of broiler chickens.

Addressing our first question, we demonstrated that the transmission of ESBL‐/pAmpC‐producing Enterobacteriaceae in the broiler production chain is more or less a summary of different transmission routes including persistence and circulation of different clonal lineages independently from antibiotic usage instead of a direct vertical top‐down transmission along the broiler production chain. Second, the occurrence of high numbers of ExPEC VAGs seemed to contribute the colonization process. However, regarding the avian host, not only high numbers of VAGs but also a certain set of virulence traits seemed to contribute this colonization. This needs to be further investigated. Finally, high numbers of isolates producing curli fibres and cellulose were detected which support the survival of resistant strains in the environment of the broiler production chain.

## Experimental procedures

### Bacterial isolates

ESBL‐/pAmpC‐producing *E. coli* originate from individual animal samples (cloacal swabs) and environmental samples (swabs and housing environment) of different stages of the broiler production chain (parent flock, hatchery, fattening farm) collected in Germany during the years 2014 and 2015 (Daehre *et al*., 2017; Projahn *et al*., 2017).

**Figure 4:**
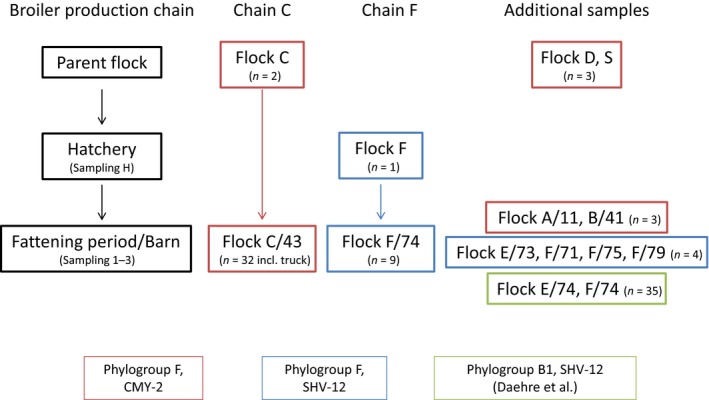
Origin and number of *E. coli* isolates used in the study for transmission investigations using WGS. Isolates of the same genetic characteristics (phylogroup + ESBL/pAmpC gene) are coloured in red, blue and green, respectively. Additional samples from the fattening period of flock F (F/71, F/75, F/79) were collected at the same time from other barns within the same farm as F/74. Isolates from flock E/74 and F/74 were only investigated concerning VAGs and Biofilm production.

In total, 54 isolates were selected on the basis of their phylogroup and the *bla* resistance gene for detailed genomic analyses using WGS to determine possible transmission events along the broiler production chain (Fig. 4; Table S1).

*Escherichia coli* of phylogroup F encoding a CMY‐2 cephamycinase were collected from samples of the parent flock C (*n* = 2) as well as from the respective fattening flock C (barn 43; *n* = 31) and the transportation truck (*n* = 1) of fattening flock C from the hatchery to the fattening farm. Additionally, strains showing the same characteristics were isolated from screening samples (faeces or boot swab), collected at different time points within the study, of parent flock D (*n* = 2) and parent screening flock S (*n* = 1) and the fattening flocks A (barn 11; *n* = 1) and B (barn 41; *n* = 2).

Isolates of phylogroup F coding for a SHV‐12 beta‐lactamase were determined from the investigation of the broiler chain F. Here, an environmental gauze swab from the hatchery (hatching of the flock F chicks; *n* = 1) as well as different samples from the fattening period of flock F (barn 74; cloacal swabs, gauze swabs, environmental swabs, housing environment; *n* = 9) were analysed. Additionally, isolates of phylogroup F encoding a SHV‐12 beta‐lactamase from boot swabs of fattening barns E/71, F/73, F/75 and F/79 were included in the comparative genome analyses. These samples originate from other flocks fattened at the same time at the same farm as flock F or E, respectively, but in different barns.

*Escherichia coli* isolates of phylogroup B1 harbouring a *bla*_SHV‐12_ gene (*n* = 35) were included in the study for the investigation of VAGs. These isolates were derived from two broiler flocks subsequently fattened in the same barn (flock E/74 and F/74) and from an additional gauze swab from the transportation truck of flock F (Daehre *et al*., 2017).

### Whole genome sequencing

Genomic DNA preparation was performed using the MasterPure™ DNA purification kit (Epicentre, Illumina) according to the manufacturer's instructions. Whole genome sequencing was performed using the Nextera XT Kit for library preparation and the Illumina MiSeq Reagent Kit v3 (300‐bp paired‐end sequencing with 50–100× coverage). The NGS tool kit (Patel and Jain, 2012) was used for quality control of the sequence read data. High‐quality reads were *de novo* assembled into contiguous sequences using CLC Genomics Workbench 8.0 (CLC bio, Aarhus, Denmark), and annotation of draft genomes was performed with RAST server (Aziz *et al*., 2008).

### Phylogenetic analyses

Whole genome data were used for *in silico* determination of MLST types using the CGE Bacterial Analysis Pipeline (Thomsen *et al*., 2016).

The bacterial core genome and the number of SNP differences were calculated between isolates of the same MLST type using Harvest suite 1.0 (Treangen *et al*., 2014) and mega 7.0 (Kumar *et al*., 2016). Phylogenetic trees were constructed using a pairwise distance matrix and the neighbour‐joining algorithm (mega 7.0, 1000 bootstraps) based on the number of calculated SNP differences between the isolates.

### Virulence‐associated genes

The presence of 89 virulence‐associated genes (VAGs) in the genomes of all 54 investigated isolates (Fig. 4) was checked using an automated in‐house blast search. VAG analyses were also performed for additional 35 isolates of the ST‐2307 (fattening flock E), which originate from samples collected from the same barn as fattening flock F but from the previous fattening round (Table S3), to overall investigate samples from fattening flocks with differing ESBL/pAmpC prevalence (flock C: 73.1%, flock E: 26.4%, flock F: 5.7%). Strains were previously investigated concerning a possible horizontal transmission scenario (Daehre *et al*., 2017).

### Phenotypic characterization

To determine the expression of biofilm‐associated extracellular matrix components (cellulose, curli fimbriae), a macrocolony assay (Schaufler *et al*., 2016a) was applied to thirteen selected isolates from the whole genome approach (at least three isolates of each determined MLST type) as well as to 38 *E. coli* and four *E. fergusonii* isolates of the studies of Projahn *et al*. and Daehre *et al*. representing all the different detected isolate profiles (phylogroup + *bla* resistance gene) determined during the investigation of seven broiler production chains (Tables S1 and S2).

## Conflict of Interest

The authors declare no conflict of interest.

## Supporting information


**Table S1.** Isolates used in the study for investigation of vertical transmissions, occurence of virulence associated genes and production onf curli.
**Table S2.** Isolates of the second set investigated by a macrocolony assay. Isolates comprise all detected genotypes (phylogroup+bla resistance).
**Table S3.** Numbers and occurrence of the 80 investigated virulence associated genes.
